# Cholera Fantasies

**Published:** 1835

**Authors:** 


OF THE

### MEDICAL AND PHYSICAL SCIENCES:

FOR APRIL, MAY AND JUNE, 1835.

### ESSAYS AND CASES.

### Art. I.—Cholera Fantasies. By the Editor.

Whether the following speculations are worth reading, it is impossible for any one to know, till he has done it. If familiar with the countless numbers of pathological hypotheses of cholera, which have followed in the train of that epidemic, he will at once perceive that they have as slender claims to originality as he may think they have to sound philosophy. It may be asked, then, why publish them? Not feeling bound to answer this question, we shall pass it by and proceed to state our premises, some of which may be thought as fit to premise any other, as a cholera speculation.

1. When the disease prevailed in Baltimore, it was observed in certain manufactories of sulphuric acid, that the rate of the oxygenation of the sulphur was exceedingly diminished.*

*This fact was communicated to us by Dr. W. Byrd Powell, who stated that he was assured of it by two extensive manufacturers.

2. Oxygen is necessaay to the elimination of carbon from the blood.

3. If the carbon be not excreted from the system, but accumulates in the blood, that fluid, of course suffers deterioration.

4. This deterioration will manifest itself to our senses, by a darker—a more carbonaceous hue.

5. In whatever manner a change in the component parts of the blood is brought about—whether, by the retention of matter which should be eliminated, or by introductions from without, the harmony between that fluid and the vital properties of the interior tunic of the vascular system, is destroyed.

6. When foreign substances even of a mild kind, whether gaseous or liquid, are injected into the veins, and made to mingle with the blood, they produce great prostration of the vital powers, under which the animal sinks immediately without their being eliminated; or vomiting and copious watery purging ensue, when he either recovers or dies.

7. Whenever and wherever the cholera is epidemic and highly malignant, individuals previously in apparent health, occasionally fall prostrate and soon expire, without any great evacuation, and without the symptoms of acute visceral inflammation.

8. More commonly, the first symptom of the disease, is a copious secretion from the alimentary mucous membrane, of a fluid which, when uncombined with the recremental matters existing in the stomach and intestines, resembles the serum of the blood, and has been proved to be that element of the sanguineous mass but little altered by its transudation through the membrane.

9. If this discharge be arrested in its early stages, the patient in general speedily recovers, especially if perspiration be promoted.

10. Should it continue, the patient quickly passes into exhaustion and collapse, though with much greater disturbance of the functions than attends mere hæmorrhage.

11. In the latter stages of fatal cholera, and after death, various parts of the surface of the body assume a blue or sooty complexion.

12. From the time the effusion has become copious, the heat of the body greatly diminishes, evincing, with other symptoms, the absence of fever.

13. *Post mortem* examinations have revealed but few traces of inflammation.

14. Weak saline tepid injections into the veins, to supply the place of the lost serum, have frequently revived the patient, greatly improved his condition and feelings, and sometimes effected a cure.

Let us now proceed to reason on these, and other facts of a like kind which need not be premised.

We commence with the *assumption*, (resting however on the first fact enumerated, but for which we admit we cannot vouch,) that the condition of the atmosphere when and where cholera prevails, is one which, from a deficiency of oxygen, is unfavorable to the due elimination of carbon from the blood. Let this, for the sake of argument, be admitted, and we proceed to inquire into the consequences.

If the blood be not adequately decarbonated, it of course becomes changed in its constitution; and many facts familiar to the profession, would seem to justify the conclusion, that this change is one which enfeebles the vital powers of the solids; a state which we constantly find present in cholera, even in its early stages. The humoral contamination continuing, the conservative powers of the system are invoked to relieve the internal parieties of the vessels from the offending materials, precisely as those powers are exerted to expel offensive matter from the ærial or alimentary passages. The depuratory outlet of the sanguineous system, as when foreign matters are injected into the veins, or the kidneys cease to secrete, or, sometimes, when dropsical fluids have been absorbed, is the intestinal membrane. In all of these cases, this membrane is excited into increased secretory or permeatory action, by the irritating influence of the peccant matter in the blood, and not by the action of any thing lodged on its surface from without, and under this irritation from within the eliminating discharge is set up. For some time, while this discharge is going on, the patient does not feel particularly uncomfortable; and, if not alarmed by moral influences, is apt to think himself but little indisposed; a state of feeling incompatible with the theory of gastro-enteritis, spinitis, encephalitis, or any other original inflammation. At length, however, he begins to feel exhausted in his muscles of locomotion, and the powers of the heart are reduced, precisely as if he had lost a large quantity of blood, so gradually as not to produce syncope. He lies down, and his pulse is weak and empty; the heat of his system is reduced, and all his functions are performed languidly. The secretion of urine, bile, and perspiration is greatly diminished, if not suspended; and all the organic energies of the system seem to be monopolized by the alimentary membrane, which is *diligently occupied* in conveying off the serum of the blood,—for the *purpose* of relieving the vessels from the impress of the irritating carbonaceous element. The work, however, like much of that performed by the *vis medicatrix naturæ*, produces no useful result, for, *as we may presume*, the carbonaceous matter chiefly remains behind, in connection with the fibrin and red globules; which, deprived of a large portion of the serum, and contaminated with the retained carbonaceous matter, constitute a fluid very different from healthy blood; which circulating throughout the vital organs, irritates and enfeebles them still more. Thus by the combined influence of a diminution in the quantity, and a change in the quality of the blood, the stage of collapse is at length produced; and although in this subdued state of the circulation, as in the debility from hæmorrhage, the discharges may cease, still the vital organs, supplied with but little blood, and that little altered in its qualities, may not be able to recover from their exhaustion, and at length cease to perform their functions. This condition of the circulation may fairly be considered as utterly unfavorable to the development of animal heat, which is well known to be in most cases very small.

The same state of the blood—a languid movement, thick consistence and carbonaceous distemperature—explains the stagnations in different parts of the capillary tissue, and the blue or purple hue which they exhibit before, and which is often much increased after death.

Finally, as to the cramps and spasms of the muscles of the limbs and sometimes of the heart, they may arise in part from the depletion, in part from the brain and the spinal marrow and the heart; being irritated by the dark and peccant fluid which circulates through or stagnates in their substance; and in part from the impress of the infiltrating serum upon the surface of the membrane through which it has passed. We are aware, that it has been often denied that secreting surfaces are ever irritated by the fluids which the tissues beneath throw upon them. But where is the proof of this fact? Who has not met with ulcers, in which the accumulation of a vitiated purulent, or a sanious fluid, under the dressings, gave great uneasiness? Do we not constantly meet with cases of ordinary cholera morbus, where the vomiting and diarrhœa appear to be the effect of the reaction of the bile upon the mucous membrane, although that membrane is its natural recipient? When the eye is inflamed, and tears accumulate beneath the lids, they greatly increase the anguish of the patient, who always expresses his gratification at their escape. In common diarrhœa and cholera, the patient generally feels much more comfortable after a discharge, and the same remark is true of epidemic cholera. Now do not these facts indicate, that secreting surfaces may be irritated by their own vitiated secretions? We believe they do, and shall continue to think so, till stronger proofs to the contrary, than we have yet seen shall present themselves.

These views of the pathology of cholera, if the first proposition,—an altered and carbonaceous state of the blood were established—would, it appears to us, explain more of the phenomena of the disease than those theories which recognize a primary irritation of the gastro-enteric mucous membrane, by an agent applied to it from without; but if called on to assign the mode in which the supposed state of the atmosphere is produced, reproduced, and extended, we can no more do it, than we can explain the origin and propagation of any other of the numerous atmospheric agents, to which the epidemic has been confidently ascribed. In the absence of all positive and accurate knowledge in this branch of ætiology, we must be content to observe the symptoms and morbid appearances; and by their aid endeavor to determine the *modus agendi* of the unknown cause. This, we have assumed, is through the lungs or the blood, and in a negative manner. From a certain morbid condition of that fluid, results the diarrhœa, which contributes, by drawing off the serum, still further to unfit the blood from performing its functions, while all the sinister effects are augmented by a reduction in the quantity. Under these views the suspension of the other secretions than the intestinal, is secondary and consequential; and the ultimate failure of all the functions, is the effect of the organs being inadequately supplied with blood, and of what they receive being changed in its qualities.

Of course, we anticipate that all this will be denounced as humoralism. But should it be denounced because it is humoralism? We think not. On the animal kingdom might be inscribed the motto, *omnis e liquore.* All the solids are built up and out of the fluids; into and out of which various matters must continually pass. To say that they never become contaminated, without a previous disease of the solids, is, we humbly conceive, quite gratuitous. It is undeniable that disease in the solids affects the fluids, and where is the warrant for declaring these morbid influences not reciprocal? We are for no *exclusive* system of solidism or humoralism. Man is a compound of liquids and solids, both are necessary in his physiological condition, and both must particpate in his pathological state. If the fluids undergo a change they irritate the containing solids, raising or depressing and perverting their actions and powers, and generally occasioning some great excretion, as a means of depuration. Thus in the decline of fevers, we have *critical* perspirations and diarrhœas to carry off the sanguinous impurities, and in cholera profuse serous discharges for a similar purpose, with this difference, that in the former case the peccant matter is the effect of the fever, in the latter the remote cause itself, or something absorbed or retained in the blood, is the agent, without any intervening morbid action of the solids.

Let us, by a reference to several remedies, see how far these speculations can guide us in practice.

1. Bloodletting. Should the condition of the blood, which we have *assumed* to exist, occur in a person of sanguine temperament, and not lead to profuse diarrhœa, it may awaken more or less inflammatory action or a general phlogistic diathesis, in which case venesection is beneficial, by diminishing the general mass of contaminated blood, and by predisposing to increased healthy secretion from the glandular apparatus generally, whereby the offensive element is eliminated. But should the serous effusion have been copious, bloodletting is not required, for, (as we assume,) there is no inflammation to subdue, and it is even prejudicial, because it increases the vascular inanition; and who that has practised in the epidemic can venture to affirm, that in this condition he has found any beneficial results from venesection? Still less we believe has it been serviceable in the stages of collapse, if that condition has been preceded by copious secretion, instead of resulting from the immediate impress on the heart and brain and other vital organs of the altered blood.

2. Emetics. When these have done good, it was generally in the early periods of the disease, and they seem to have acted by putting aside the secretory operation going on in the membrane below, and by determining upon the surface of the body—in other words, by arresting that discharge which must be stopped or the patient dies. On a similar principle they restrain hæmorrhages. It is worthy of remark, that those which are combined with stimulants are most efficacious, which is what might be expected on the hypothesis we are advocating, but not on that of gastro-enteritis. In *advanced* stages of the disease, we have not seen them of any substantial benefit, except when composed of the most stimulating ingredients, such, indeed, as are inadmissible in gastro-enteritis.

3. Cathartics. These are only useful in the early stages, when, as in other cases of incipient diarrhœa, they substitute an action of their own, for the morbid action previously existing, and thus arrest the inordinate secretion.

4. Mercurials. These have been given to excite the function of the liver. It has been assumed that a failure or suspension of this function was the cause of the diarrhœa, but our hypothesis makes it the *effect*. Still there is *reason* in the mercurial practice. Calomel may be capable, to some extent, of setting aside the secretory action of the mucous membrane, and thus arresting the discharge; and by increasing or restoring the secretion of bile, it may arrest that of serum, for among a group of organs which are overthrown in their functions, if we succeed in rectifying one, the others from association are often righted up.

5. Saline Draughts and Venous Injections. The former might do good if they were absorbed; but there is no evidence that during an attack of cholera, much absorption takes place; and when they are thrown into the veins, they have too little resemblance to the lost serum to supply its place, and act too much in bulk upon the solids before they can be mixed with the blood.

6. Internal Stimulants. These may do good during the stage of secretion, by superseding the morbid secretory action, and afterwards by supporting the powers of the system. But while their efficacy has not been great it has been too much to be reconciled with the theory of gastro-enteritis.

7. External Stimulants. These have done good, by inviting the blood from the centre to the circumference of the system—thus restraining the discharge—and by contributing to correct its enervating influence.

8. Ice. The beneficial effects of ice in cholera are probably of the same kind as in some forms of hæmorrhage; and limited to a restraining of the discharge, to which, however, it is often inadequate.

9. Narcotics. If a mere nervous irritation, independent of a morbid condition of the blood, was the essential circumstance in cholera, narcotics ought to do far more good than they have yet done; and if inflammation existed they should have done much greater mischief. Their effects seem to us to be auxiliary and palliative—generally acceptable to the patient, and as in hæmorrhage restraining to the discharge.

10. Astringents. On the hypothesis to which we are devoting an idle hour, astringents might be considered as useful. When thrown into the lower bowels they sometimes perhaps restrain the external evacuation. When taken into the stomach they are too often like our other remedies, immediately ejected. If made to pass the pylorus in large quantities, they might be expected to do good, provided they were capable of restraining the exudation of serum through the membrane. In a late number of the Journal we were enabled to give an encouraging account of one of this class of medicines—the acetate of lead. It seemed to act by arresting the secretion, which is the first and great object to be accomplished. It is not, therefore, adapted to the advanced stages of the disease. To be effective it should be given in ten grain doses with opium, and repeated every one, two, or three hours, according to the profuseness of the discharge.

11. Oxygen Gas. On the hypothesis of accumulated carbon in the blood, the application of this gas ought to do good. At this moment we do not recollect the results which have been obtained by those who have given it a trial. It might be used, first by inhalation; second, by immersion of the body of the patient in an atmosphere of the same gas; third, by giving him draughts and enemata of cold water, in which the gas has been condensed by a forcing pump. All of these modes might be practiced in cities and hospitals, but would be impossible in the country.

---

The foregoing pages contain the substance of some extempore remarks in the *Cincinnati Medical Society*. They have been written down, merely to see how far a humoral theory, which we do not claim as our own, can be made to explain the phenomena of cholera. Since they were prepared for the press, we have met with a paper by Prof. Horner in the May No. of the American Journal, on the morbid anatomy of this disease, some account of which, by Prof. Gross, will be found under our head of Reviews, in this number.

The morbid appearances which Dr. H. has described, differ in some respects from those detailed by other observers, but, as far as they relate to the alimentary canal, seem to us to be utterly insufficient, to explain the early mortality of this disease; and, negatively, go to confirm us in the opinion, that we must look elsewhere, than to the digestive tube, for those lesions which occasion death. But in what organ do they exist? Certainly not in the nervous centres, the heart, or the lungs. We are driven then, it would appear, to the blood; as in the reduced and altered condition of that fluid, and there only, do we find an adequate cause, for the death of the patient. When the entire mass is greatly reduced in quantity, inspissated, and black, both in the arteries and veins, in the left as well as the right cavities of the heart, why should we hesitate to ascribe the fate of the patient to hæmatic alterations? Is not a certain amount of decarbonated blood indispensable to the support of the phenomena of life? and if we find it wanting should we not expect those phenomena to cease? Should future researches reveal to us organic changes in the solids, of a kind not hitherto brought to light, the fact of a fatal lesion of the blood would still continue, and to it these very changes might perhaps be ascribed. Thus to the hæmatic vitiation and serous exfiltration, we might attribute all the morbid appearances found in the mucous membrane of the alimentary canal, much more rationally, it appears to us, than to reverse the proposition, and derive the former from the latter.

In 1832 we had occasion several times to quote from Mr. Greenhow, of New Castle, Eng., one of the most judicious writers on cholera which that country has produced. A recent number, (April, 1835,) of the Edinburg Medical and Surgical Journal contains a paper from that gentleman, giving his latest experience in the treatment of the disease; and although he is a believer in the doctrine of a primary impression on the mucous membrane of the alimentary canal, he evidently inclines to the opinion, that life is destroyed by the influence on the blood, of the excessive exfiltration through that membrane, and prescribes accordingly. To restrain the discharge, he proposes a medicine, which we have not used, and which, *a priori*, we should think inferior to the acetate of lead. We shall, however, conclude this article with such an extract from his paper, as will make known his views, and enable such of our readers as think well of his remedy, to employ it in his own way. The extract relates to that *stadium* of the disease, which is characterized by serous discharges.

"In the stage of rice-water dejections, which, properly speaking, is the first stage in which we can with certainty pronounce the disease cholera, I have tried nearly all the plans which have at various times been brought forward as successful, and I cannot say that I was satisfied with the result of any one remedy. A small proportion of my patients recovered, it is true; but it always remained a matter of doubt upon my mind, how far the means employed were conducive to that end; and I naturally considered what remedy was most likely to exert a control over a discharge which was evidently draining the system of the fluid necessary to carry on the functions of life; and, considering the case as one of passive hæmorrhage, I at once decided upon trying the sulphuric acid, from its well known powers of coagulating albumen, as well as acting as an astringent in closing the relaxed mouths of the exhalent. The first case in which I tried it, after the first dose the stools changed their character, being a mass of coagulated albumen; after the third dose the purging ceased, and at the end of fourteen hours the patient passed a feculent motion mixed with albuminous flakes, and the patient recovered without experiencing any check. I have not detailed the case more fully, as it appeared at the time in the London Medical Gazette. Since then I have only had an opportunity of trying it in two cases, in both of which it arrested the rice-water dejections in a precisely similar manner, leaving no doubt as to the effect being produced by the acid. One of the last mentioned patients had a second attack of rice-water purging when in a state of convalescence, from committing an error in diet. He was in fact a confirmed sot. The complaint was a second time arrested by the acid, but the man died a month afterwards, his digestive organs being destroyed by his long-continued habits of intemperance. The form in which I have hitherto exhibited the acid is the following:

℞. *Infus. Caryoph,* ℥vi.; *Acid. Sulph. dil.* ʒii.; *Tinc. Opii.* gtt. xxx.; *Sacc. Pur.* ℥ss. *M. Cap.* ℥i. 6m. *hor.* and at longer intervals when the purging is restrained."

The delay of our publishers, enables us to announce the resuscitation of the epidemic in the Valley of the Mississippi, constituting its fourth annual invasion. As early as the month of May, perhaps earlier, it appeared in various parts of the states of Mississippi and Tennessee, and in the succeeding month was revived in Missouri, Illinois, Indiana, Kentucky, and Ohio. Wide however as has been its geographical range, the mortality has not been great, compared with that of previous years, and but few places have been visited. Several towns, as Memphis, Tennessee; Madison, Indiana; and certain circumscribed spots, in the country, have experienced malignant and fatal visitations, while the surrounding inhabitants entirely escaped, or but a single violent case now and then occurred. Cincinnati has suffered in so slight a degree, as not to experience the least disturbance of social and commercial relations. Indeed it has been sporadic only; and not a few of the cases have been in persons who came from the country. At present, July 20, the city and surrounding country, as far as we know, are quite free from all epidemic distemperature. In fact the spring and summer so far, have been among our healthiest; and, we may add, our coolest and rainiest. For many years, we do not recollect a period of six or eight weeks before and after the summer solstice, in which a greater number of copious showers occurred, or in which the heat was more temperate.

